# Development and Characterization of Monoclonal Antibodies to the 32 kDa Viral Attachment Protein of Lymphocystis Disease Virus and Their Neutralizing Ability in Vitro

**DOI:** 10.3390/ijms19092536

**Published:** 2018-08-27

**Authors:** Ying Zhong, Xiaoqian Tang, Xiuzhen Sheng, Jing Xing, Wenbin Zhan

**Affiliations:** 1 Laboratory of Pathology and Immunology of Aquatic Animals, KLMME, Ocean University of China, Qingdao 266003, China; 18300213280@163.com (Y.Z.); tangxq@ouc.edu.cn (X.T.); xingjing@ouc.edu.cn (J.X.); wbzhan@ouc.edu.cn (W.Z.); 2 Function Laboratory for Marine Fisheries Science and Food Production Processes, Qingdao National Laboratory for Marine Science and Technology, Qingdao 266071, China

**Keywords:** lymphocystis disease virus, monoclonal antibody, viral attachment protein, neutralization, flounder (*Paralichthys olivaceus*)

## Abstract

In previous research, a 32 kDa protein in lymphocystis disease virus (LCDV) was identified as viral attachment protein (VAP) that specifically interacted with the 27.8 kDa cellular receptor from flounder *Paralichthys olivaceus* gill (FG) cells, and the recombinant VAP (rVAP) was expressed in *Escherichia coli* strain BL21 (DE3). In this study, monoclonal antibodies (MAbs) against 32 kDa VAP are produced by immunization of BALB/c mice with the rVAP. Seven hybridoma secreting MAbs were screened by enzyme-linked immunosorbent assay, five of which designated as 1C6, 1C8, 3B5, 3D11 and 3H10 are cloned by the limiting dilution method, depending on the strongly positive results of ELISA. Western blotting analysis shows that the five MAbs can specifically react with the 32 kDa protein of LCDV and the purified 50 kDa rVAP, and the subtype of the MAbs is identified as IgG. Immunofluorescence results demonstrate that the specific fluorescence signals for LCDV appear in the cytoplasm of FG cells at 24 h post LCDV infection. Neutralization assay results indicate that pre-incubations of LCDV with the five MAbs can significantly decrease the LCDV copy numbers and delay the development of the cytopathic effect in FG cells, revealing that the five MAbs can neutralize the LCDV particles and block viral infection in vitro. The neutralizing MAbs against 32 kDa VAP would be useful for the study on the LCDV–host interaction and might be promising inhibitors of LCDV infection in fish.

## 1. Introduction

Lymphocystis disease (LCD) is a viral disease occurring in marine, brackish and freshwater teleost fish with worldwide distribution [1], which is characterized by the appearance of pearl-like nodules on the skin, fins, tail and the internal organ of the affected fish [2]. Since the first outbreak of the disease in Shandong Province, China, this disease has spread to coastal provinces and affected more than 10 fish species, causing huge economic losses to Chinese aquaculture industry [3]. The causative agent of LCD is lymphocystis disease virus (LCDV), a large icosahedral DNA virus in the genus *Lymphocystivirus* of the family *Iridoviridae* [4], with double-layered capsid, an outer envelope and a fringe consisting of fibril-like external protrusions [2,5,6]. The researches have investigated the gene encoding, the major capsid protein and the genetic diversity of LCDV [7,8], virus detection [9], lymphocystis cell formation [10], and the complete gene sequences of two LCDV strains, LCDV isolated from flounder *Platichtys flesus* in Europe (LCDV-1) and LCDV isolated from *Paralichtys olivaceus* in China (LCDV-C) [11,12]. Notably, a 27.8 kDa membrane protein from flounder (*P. olivaceus*) gill (FG) cells has been recently identified as a cellular receptor to mediate LCDV binding and infection [13,14,15], and a 32 kDa envelop protein of LCDV, encoded by the open reading frame (ORF) 038 gene in LCDV-C, is found as a viral attachment protein (VAP) to interact with the 27.8 kDa receptor protein [16]. However, no effective methods are developed for the prevention and cure of fish lymphocystis disease [17,18], and the molecular mechanism underlying LCDV infection and pathogenesis, especially the LCDV–host interaction, also needs to be further clarified.

Antibodies that bind to and inactivate the virions are generally referred to as neutralizing antibodies, which play an essential part in antiviral immunity and are instrumental in preventing or modulating viral disease [19,20]. In our previous research, based on the ORF 038 gene encoding the 32 kDa VAP of LCDV, *Escherichia coli* strain BL21 (DE3) containing recombinant plasmid pET-32a-ORF038 of LCDV was constructed, and the recombinant VAP (rVAP) protein was expressed; moreover, it was evidenced that polyclonal antibody against rVAP could neutralize LCDV infection to FG cells [16]. Therefore, the development of monoclonal antibodies (MAbs) against 32 kDa VAP would be useful for studies of virus–receptor interaction and provide a new way for the prevention and cure of lymphocystis disease in fish. In this study, the MAbs against the 32 kDa VAP were obtained by immunization of BALB/c mice with the rVAP, and characterized by enzyme-linked immunosorbent assay (ELISA), indirect immunofluorescence assay (IFA) and western blotting. Furthermore, neutralization assay was conducted to analyze the ability of the VAP MAbs to block LCDV infection.

## 2. Results

### 2.1. Expression, Purification and SDS-PAGE Analysis of Recombinant Viral Attachment Protein

**Figure 1:**
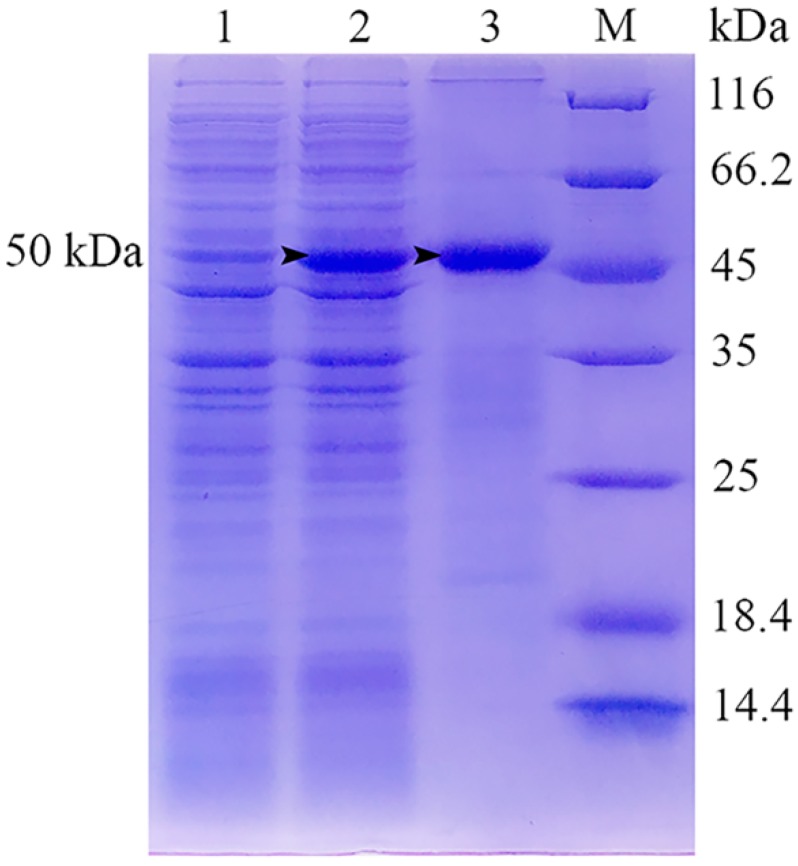
Expression, purification and SDS-PAGE analysis of recombinant viral attachment protein (rVAP) expressed in *E. coli* strain BL21. The proteins were visualized by Coomassie brilliant blue R-250. Lane 1: rVAP before induction; lane 2: induced rVAP by IPTG; lane 3: purified rVAP; M: marker. Arrow: the induced 50 kDa rVAP and purified 50 kDa rVAP.

The *E. coli* strain BL21 that contained recombinant plasmid pET-32a-ORF038 was cultured in the LB medium and the expression of rVAP was induced by isopropyl-β-D thiogalactopyranoside (IPTG). SDS-PAGE showed the induced protein band was enhanced at 50 kDa, which was consistent with the prediction of a 32 kDa VAP plus a His-tagged protein of 18 kDa (Figure 1, lane 2). The rVAP was purified by HisTrap Ni–NTA affinity chromatography, and the purified rVAP was dialyzed and freeze-dried. SDS-PAGE indicated that the purified protein was 50 kDa, and the protein band was single and no other protein was shown (Figure 1, lane 3), which could be used for the preparation of MAbs.

### 2.2. Production, Screening and Subtype Identification of Anti-32 kDa Viral Attachment Protein Monoclonal Antibodies

**Figure 2:**
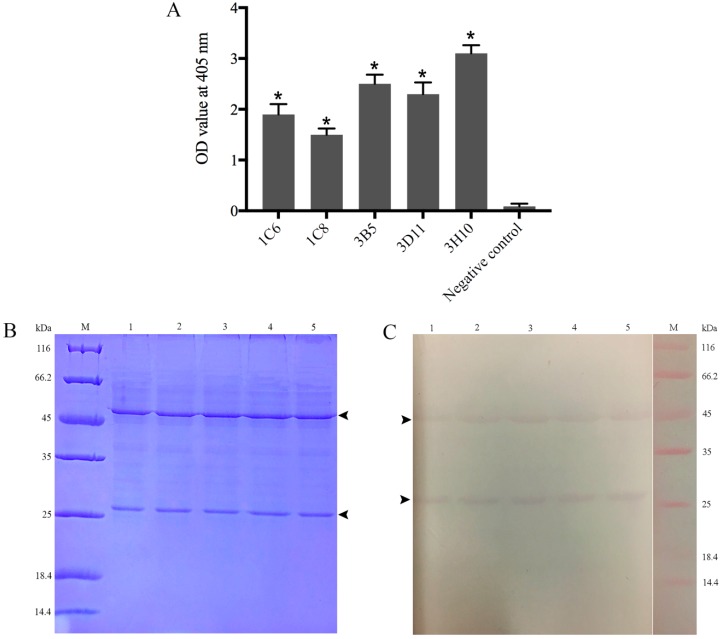
Screening and subtype identification of anti-32 kDa viral attachment protein (VAP) monoclonal antibodies (MAbs). (**A**) The five anti-32 kDa VAP MAbs reacted with the rVAP by ELISA. The culture supernatant of MAbs against white spot syndrome virus (WSSV) instead of primary antibody was used as a negative control. Error bars represent standard deviations (SD). Asterisk denotes significant differences comparing with the negative control (*p* < 0.05). (**B**) SDS-PAGE analysis of the purified five VAP MAbs. (**C**) The five anti-32 kDa VAP MAbs were identified as IgG subtype by western blotting. Lanes 1–5: MAbs 1C6, 1C8, 3B5, 3D11, 3H10. The arrows show the 50 kDa heavy chain and 27 kDa light chain of IgG.

The spleen cells of immunized mice were fused with myeloma cells under aseptic conditions. About two weeks later, supernatants of the fusions were first screened by ELISA, and twenty positive hybridomas in 96-well plates were transferred into 24-well plates and screened again by ELISA. Ultimately, seven hybridomas secreting anti-32 kDa VAP MAbs were selected, five of which designated as 1C6, 1C8, 3B5, 3D11 and 3H10 were strongly positive based on their high absorbance value (OD > 1.0) and cloned by the limiting dilution method (Figure 2A). The negative control using culture supernatant of MAbs against white spot syndrome virus (WSSV), instead of primary antibody, showed a very low background (OD < 0.1).

To identify the subtype of the five MAbs, supernatant of the five MAbs was subjected to SDS-PAGE, transferred to polyvinylidene difluoride (PVDF) membranes separately, and then incubated with alkaline phosphatase (AP)-conjugated goat-anti-mouse immunoglobin (Ig). Results showed that the five MAbs were all IgG-type with the heavy chain of about 50 kDa and the light chain of about 27 kDa (Figure 2B,C).

### 2.3. Reaction Abilities of Anti-32 kDa Viral Attachment Protein Monoclonal Antibodies

**Figure 3:**
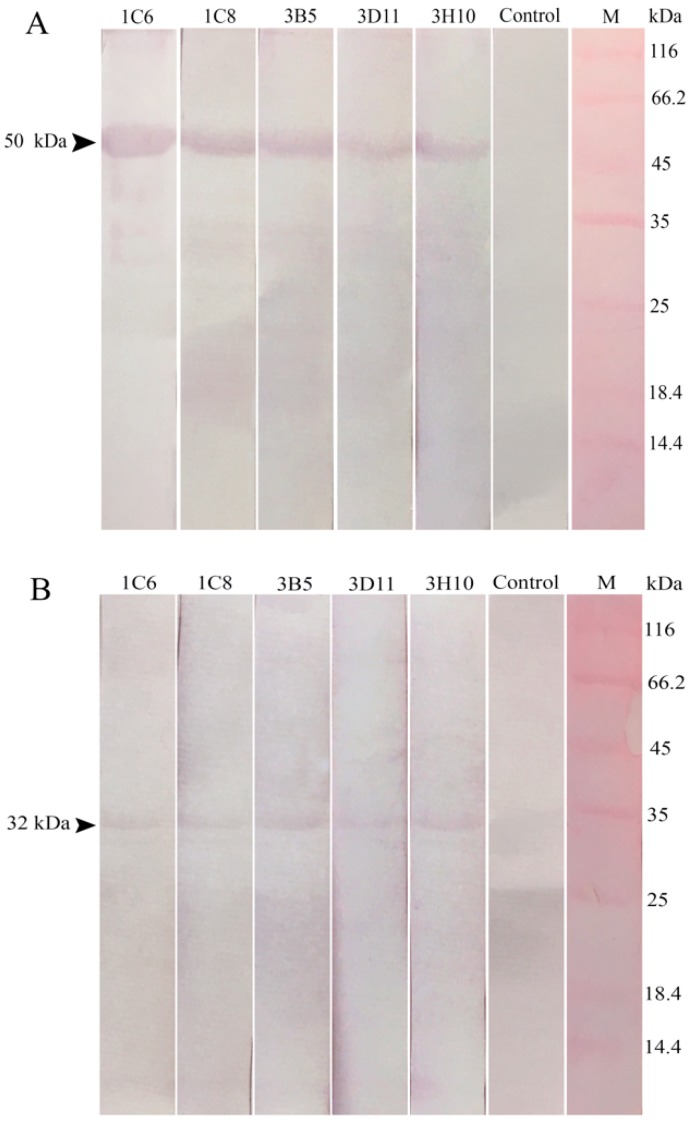
The reaction abilities of the five anti-32 kDa VAP MAbs by western blotting. (**A**) The reactions of the five MAbs with 50 kDa rVAP. (**B**) The reactions of the five MAbs with 32 kDa protein in lymphocystis disease virus (LCDV). Control: culture supernatant of MAbs against WSSV, instead of the five MAbs against 32 kDa VAP, as a negative control. M: marker. Arrow: the indicated 50 kDa rVAP and 32 kDa VAP in LCDV.

Western blotting was carried out for analyzing reaction abilities of the five anti-32 kDa VAP MAbs using the 50 kDa rVAP and purified LCDV particles. Results showed that supernatants of the five MAbs could specifically react with the 50 kDa recombinant protein (Figure 3A) and the 32 kDa protein in LCDV (Figure 3B). The negative control using culture supernatants of MAbs against WSSV, instead of primary antibody, showed no bands (Figure 3, lane control). These results suggested that the five MAbs targeted linear epitopes within the LCDV protein.

### 2.4. Lymphocystis Disease Virus Detection in Flounder *(*P. Olivaceus*)* Gill Cells by Anti-32 kDa Viral Attachment Protein Monoclonal Antibodies

**Figure 4:**
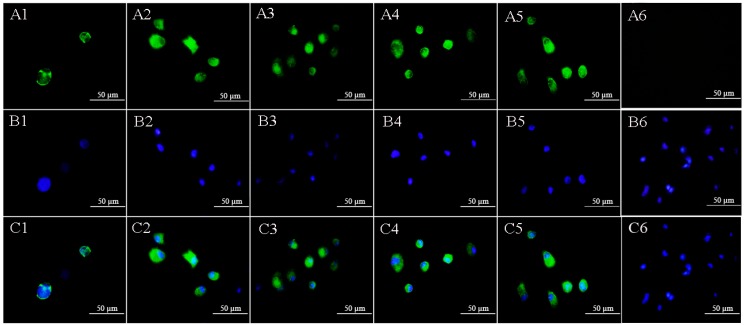
Immunofluorescence assay (IFA) of flounder *Paralichthys olivaceus* gill (FG) cells after LCDV infection using the five MAbs. (**A1**–**A5**): Immunofluorescence staining of LCDV using MAbs 1C6, 1C8, 3B5, 3D11 and 3H10, respectively; **A6**: FG cells without LCDV infection served as a negative control. (**B1**–**B6**): Cell nuclei were stained in blue by DAPI. (**C1**–**C6**): Superpositions of A1 and B1, A2 and B2, A3 and B3, A4 and B4, A5 and B5, A6 and B6, respectively. The scale bars are 50 μm.

The FG cells were seeded on cover slips and inoculated with LCDV particles, and the supernatants of the five MAbs were used to detect LCDV in FG cells after 24 h. Results showed that all the five MAbs reacted with FG cells and the specific fluorescence signals for LCDV particles appeared in the cytoplasm, but not in cell nuclei (Figure 4A1–A5). Cell nuclei were stained in blue by 4,6-diamidino-2-phenylindole (DAPI) (Figure 4B1–B6). No fluorescence was observed in the negative control (Figure 4A6).

### 2.5. Neutralization Ability of the Monoclonal Antibodies to Lymphocystis Disease Virus Infection

**Figure 5:**
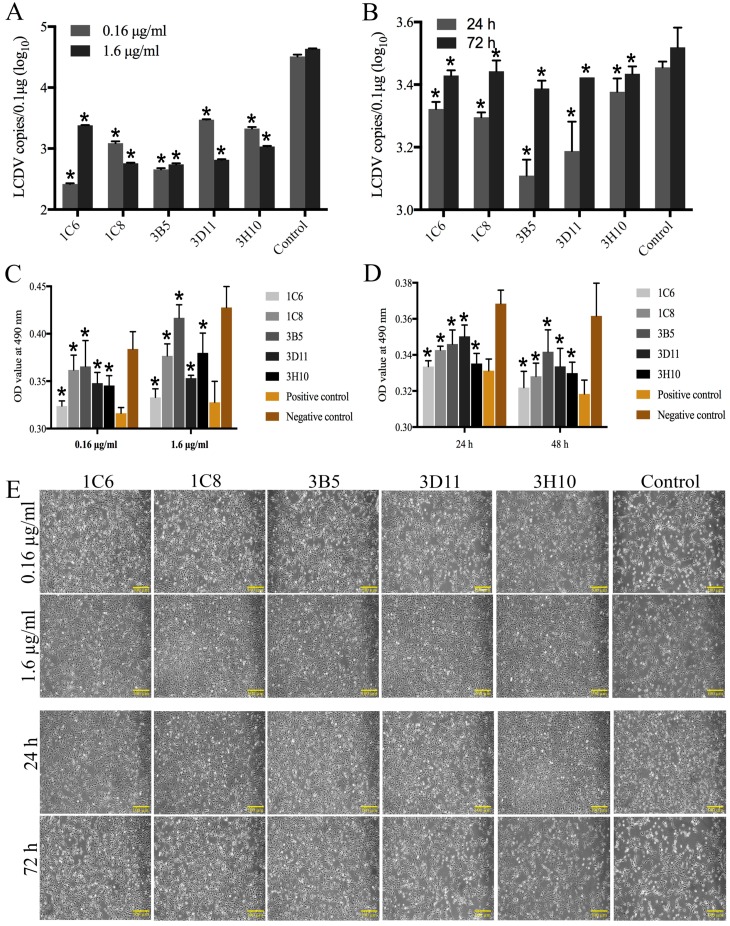
The neutralization ability of the five VAP MAbs to LCDV infection. (**A**) Virus copy numbers were decreased when LCDV was pre-incubated with the MAbs (1C6, 1C8, 3B5, 3D11, 3H10) of different concentrations as compared with the control at 48 h. (**B**) LCDV copy numbers were increased over time but lower than the control, which was pre-incubated with anti-WSSV MAbs. (**C**) The FG cell viability after inoculated with 0.16 µg/mL and 1.6 µg/mL MAbs pre-incubated LCDV. (**D**) The FG cell viability after inoculated with 1.6 µg/mL MAbs pre-incubated LCDV at 24 h and 48 h. (**E**) The cytopathic effect (CPE) in FG cells was delayed when the LCDV was pre-incubated with MAbs strain 1C6, 1C8, 3B5, 3D11 and 3H10. Control: MAbs against WSSV instead of the produced anti-32 kDa VAP MAbs served as a control, bars = 100 µm. Error bars represent SD. Asterisk denotes significant differences comparing with negative control (*p* < 0.05).

The FG cells were infected with LCDV particles, which were pre-incubated with different concentrations of purified ascites of the MAbs 1C6, 1C8, 3B5, 3D11 and 3H10. The qPCR results showed that the LCDV copy numbers in FG cells were significantly lower in all anti-32 kDa VAP MAbs-treated groups of 1.6 µg/mL and 0.16 µg/mL than in the control that was pre-incubated with anti-WSSV MAbs at 48 h post infection (hpi) (Figure 5A). In presence of 1.6 µg/mL MAbs, the LCDV copy numbers were increased over time, but significantly lower as compared with the control that was pre-incubated with anti-WSSV MAbs (Figure 5B). Furthermore, in the control that was pre-incubated with anti-WSSV MAbs, the cytopathic effect (CPE) in FG cells were obviously observed at 48 hpi, and a large number of dying cells exhibited cell rounding and detachment from the substrate, presenting some viral plaques (Figure 5E); A few plaques were also seen in all experimental groups that were pre-incubated with 0.16 µg/mL MAbs at 48 hpi; however, in presence of 1.6 µg/mL MAbs, only a few cells exhibited cell rounding, no obvious cell detachment and plaques were found at 24 and 48 hpi, but some plaques appeared at 72 hpi, indicating that the CPE was delayed. No dying cells or CPE were observed in the uninfected FG cells (data not shown). MTT cell viability assay showed that the FG cell viability after inoculated with MAbs pre-incubated LCDV was between positive and negative controls, and the cell viability of 0.16 µg/mL group was lower than the 1.6 µg/mL group, and the cell viability was reduced over time (Figure 5C,D). These results suggested that the five MAbs could neutralize LCDV infection.

## 3. Discussion

Monoclonal antibody technology has been widely used in the study of many aquatic animal viruses, such as LCDV [21], red sea bream iridovirus [22], yellowtail ascites virus [23], and WSSV [24,25]. The MAbs of aquatic animal viruses are vital for early diagnosis of viral infection [5] and the study of the virus-host interaction [14,16], virus strain typing [26], localization and functional analysis of viral structural proteins [27]. For LCDV studies, the MAbs against LCDV are produced [21] and a 27.8 kDa cellular receptor are identified through immunoprecipication using anti-LCDV MAbs [13], and thereafter, a 32 kDa envelop protein of LCDV is identified as a VAP to interact specifically with the 27.8 kDa receptor protein by using anti-27.8 kDa receptor MAbs [16]. The VAPs play important roles in virus adsorption and infection by binding to viral receptors on the host cell surface [28], and the antibodies against the VAP have neutralization ability [29]. To further study the biological roles of the 32 kDa VAP in LCDV infection, MAbs against 32 kDa VAP were produced through immunization of BALB/c mice with purified rVAP in this study.

In previous studies, five anti-LCDV MAbs were produced by our lab [21]; however, none of the five MAbs demonstrated reaction to the 32 kDa polypeptide of LCDV, which indicated that the developed MAbs against 32 kDa VAP in this study were different from the previous anti-LCDV MAbs, and they targeted different antigenic epitopes. Moreover, the five anti-32 kDa VAP MAbs could react to the 32 kDa protein of LCDV and the 50 kDa rVAP, indicating they targeted linear epitopes in LCDV. In order to identify the subtype of the developed MAbs, hybridoma cell culture supernatants were used for western blotting. The mouse IgG has a 52–54 kDa heavy chain and a 24 kDa light chain, while the IgM has a 70–80 kDa heavy chain and a 24 kDa light chain; hence, the five anti-32 kDa VAP MAbs were all IgG-type according to the western blotting results. In IFA assay, the green fluorescence signals for LCDV particles appeared in FG cells at 24 hpi, showing the existence of LCDV. All these results indicated that the developed anti-32 kDa VAP MAbs had good specificity.

There are many antigenic determinants on the surface of virus, and some of these are involved in virus adhesion and invasion. If the antibodies bind to these key parts of the virus, the virus infectivity will be weakened or even lost, that is, the neutralization occurs [30,31]. Some neutralizing antibodies of viruses have currently been developed, such as human immunodeficiency virus [32], influenza virus [33], reovirus [34] and Singapore grouper iridovirus (SGIV) [35,36]. Although little is known about neutralizing antibodies of LCDV, the reports about inactivated vaccines confirmed the existence of neutralizing antibodies against LCDV on the other side [3,37]. In this study, pre-incubation of LCDV with the produced anti-32 kDa VAP MAbs 1C6, 1C8, 3B5, 3D11 and 3H10 could significantly decrease the LCDV copy numbers, delay the CPE development in FG cells, and increase the cell viability as compared with the positive control according to MTT cell viability assay, revealing that the anti-32 kDa VAP MAbs could neutralize the LCDV particles and block viral infection in vitro. Further measurement of virus titers or neutralizing titers would improve our understanding of the neutralizing activity of the MAbs, which is worth doing in the future. The neutralization abilities of the five MAbs were different, which indicated that the five MAbs might compete for different epitope; further study is needed to clarify this. Moreover, we found that the neutralization ability was not good when the concentration of anti-32 kDa VAP MAbs was too low or too high, it might be because too low concentrations of MAb were not enough to neutralize all antigenic determinants of LCDV, whereas too high concentrations caused toxicity to cells as the FG cells were shed from the culture flasks when pre-incubated with high concentrations of MAbs. Similar phenomenon was also observed in studying the blocking ability of human monoclonal antibody to hepatitis C virus E1 glycoprotein [38]. When LCDV was incubated with the same concentration of MAbs, the virus copy numbers were increased over time, but always lower than the positive control. We also tested the neutralization ability when the five MAbs were combined, but no significant difference was observed compared with single MAb (data not shown), which might be because of the improper concentrations of the MAbs or other reasons. Therefore, further studies on identification of the epitopes, the binding conformation of the five MAbs and even the functional sites of 32 kDa VAP were required to elucidate the neutralization mechanism of the five MAbs. Currently, the binding epitope for herpes simplex virus (HSV) is identified to produce neutralizing antibody, which facilitated studies of HSV entry or vaccine design [39]. Since the interaction of 32 kDa VAP with the 27.8 kDa receptor was previously confirmed to mediate LCDV attachment and entry [16], the neutralizing anti-32 kDa VAP MAbs might be promising inhibitors to block the LCDV–receptor binding as an antiviral agent. The 32 kDa VAP of LCDV [16] has been found to be cognate with SGIV VP19 [40] and Rana grylio virus (RGV) envelope protein 2L [41], and polyclonal antibodies against SGIV capsid protein VP38 and envelop protein VP39 exhibit neutralization ability in vitro [35,36]; therefore, identification of conserved epitopes would strongly promote vaccine design, and the neutralizing anti-32 kDa VAP MAbs of LCDV might also provide a new way for the prevention and cure of iridovirus disease in fish and have a potential use for LCDV diagnostic.

In conclusion, we developed and characterized five MAbs against 32 kDa VAP of LCDV, and further confirmed the neutralization ability of the MAbs to LCDV infection in FG cells. The developed MAbs were useful for further studies on the interaction of the 32 kDa VAP of LCDV with the 27.8 kDa cellular receptor, which could promote our understanding of the molecular mechanism underlying LCDV infection. In addition, neutralizing MAbs against 32 kDa VAP might be promising inhibitors of LCDV infection in fish.

## 4. Materials and Methods

### 4.1. Ethics Statement

This study was conducted strictly in line with the procedures in the Guide for the Use of Experimental Animals of the Ocean University of China. Animal experiments were approved by the Institutional Animal Care and Use Committee of Ocean University of China (Permit Number: 20151201, 1 December 2011). All efforts were dedicated to minimizing suffering of animals.

### 4.2. Cells, Virus and Proteins

The FG cells were cultured in Minimal Essential Medium (MEM, Gibco, Waltham, MA, USA) supplemented with 10% of fetal bovine serum (FBS, Gibco, Waltham, MA, USA), 100 IU/mL penicillin and 100 μg/mL streptomycin (Gibco, Waltham, MA, USA), cultivated at 22 °C with 2% CO_2_. The purified LCDV particles were prepared in the previous study and stored at −80 °C until use [16].

The *E. coli* strain BL21 (DE3) containing recombinant plasmid pET-32a-ORF038 of LCDV was constructed and stored in our lab [16]. The *E. coli* BL21 (DE3) was cultured in LB medium in exponential growth phase at 37 °C, and then, 100 mM IPTG (1:100) (Solarbio, Beijing, China) was added to the bacterium solution to induce rVAP expression. The rVAP was purified by binding buffer (100 mM Na_2_HPO_4_, 12 mM NaH_2_PO_4_, 500 mM NaCl, 8 M urea and 20 mM imidazole, pH 7.4) and elution buffer (100 mM Na_2_HPO_4_, 12 mM NaH_2_PO_4_, 500 mM NaCl, 8 M urea and 500 mM imidazole, pH 7.4) using His Trap Ni-NTA (GE Healthcare, Chicago, IL, USA) affinity chromatography. The purified rVAP was first subjected to gradient dialysate (126 mM Na_2_HPO_4_, 11 mM NaH_2_PO_4_, 50 mM NaCl, 1 mM EDTA) containing different amounts of urea (6 M, 4 M, 2 M, 0 M), reduced glutathione (2 mM) and oxidized glutathione (0.2 mM), and finally dialyzed in phosphate buffer saline (PBS) and ultra-pure water; every gradient solution was dialyzed for 12 h. Subsequently, the purified rVAP was dried by a freezing-dryer and subjected to SDS-PAGE. The protein concentration was determined by Bradford Protein Assay Kit (Beyotime, Shanghai, China) following the manufacturer’s instructions. The purified rVAP was stored at −80 °C until use. The MAbs against WSSV used as a negative control in this study was produced by our lab [24].

### 4.3. Mouse Immunization and Monoclonal Antibody Production

For MAb production, two six-week-old female BALB/c mice were used for immunization. For the primary immunization, 100 μg purified rVAP in 50 μL PBS mixed with an equal volume of Freund’s complete adjuvant (Sigma, St. Louis, MO, USA) was injected intraperitoneally to mice. Two weeks later, booster immunization was given to each mouse with the same quantity of rVAP in incomplete Freund’s adjuvant by intraperitoneal injection, followed by two booster injections with 100 μg rVAP via the tail vein at one-week intervals. Three days after the last injection, the mice were sacrificed and the spleen was removed under aseptic conditions. Myeloma cells (P3-X63-Ag8U1, P3U1) in log phase of growth were fused with spleen cells, and hybridomas were produced using the method as described in the previous studies [42,43]. Fusion of myeloma cells and mouse spleen cells were grown in 96-well microplates (Sigma, St. Louis, MO, USA) at 37 °C with 5% CO_2_. The supernatants from 96-well microplates were screened by ELISA using the purified rVAP. Positive hybridomas were cloned using the limiting dilution method for three times [44], and MAbs against 32 kDa VAP were then further screened by western blotting and IFA.

### 4.4. Indirect Enzyme-Linked Immunosorbent Assay

The MAbs screening by ELISA was performed as follows: flat-bottom 96-well microplates were coated with 5 μg purified rVAP per well and incubated overnight at 4 °C. The wells were washed three times with PBST (PBS containing 0.05% Tween-20) and then blocked with 4% bovine serum albumin (BSA, Sigma, St. Louis, MO, USA) at 37 °C for 2 h. After washed as above, supernatants from the cultured hybridomas were added as the primary antibody and incubated at 37 °C for 1.5 h. Following three washes with PBST, AP-conjugated goat-anti-mouse Ig (Sigma, St. Louis, MO, USA) diluted 1:5000 in PBS was added as a secondary antibody and incubated at 37 °C for 1 h. After washed with PBST, 100 μL substrate solution (1% diethanolamine, 0.5 mM MgCl_2_, pH 9.8) containing 0.1% *p*-nitrophenyl phosphate (*p*NPP, Sigma, St. Louis, MO, USA) was added to each well and incubated for 15 min, and absorbencies were measured with an automatic ELISA reader (Molecular Devices) at 405 nm. Culture supernatants of MAbs against WSSV, instead of the primary antibody, were used as negative controls. The positive hybridomas were cloned using the limiting dilution method. Briefly, the spleen of four-week-old female BALB/c mouse was removed under aseptic conditions, crushed and washed two times with pre-heated 1640 (Solarbio, Beijing, China) at 37 °C and centrifuged at 100× *g* for 5 min, and then the precipitate was resuspended with 20 mL pre-heated GIT medium (Nihon Seiyaku Co., Fukuoka, Japan) containing 2% HAT media supplement hybrimax (Sigma, St. Louis, MO, USA). Thereafter, the hybridoma cell suspension to be cloned was prepared, and the hybridoma cells were diluted to 10 cells/mL after counting. About 100 hybridoma cells were added to 10 mL spleen cell suspension, and the suspension was incubated in 96-well microplates, with the amount of 0.1 mL per well. The cloned positive hybridoma cells were grown at 37 °C with 5% CO_2_ and observed under an inverted microscope to mark the wells with single clonal growth about one week later.

### 4.5. Subtype Identification of the MAbs

The hybridoma culture supernatants were centrifuged at 1000× *g* for 5 min to remove cell debris, and then 20 μL supernatant was mixed with an equal volume of protein loading buffer (Takara, Japan). After denatured for 5 min in boiling water, the mixtures were subjected to SDS-PAGE (one was stained with Coomassie blue R-250, and the other was transferred to PVDF membranes (Millipore, Burlington, MA, USA)), and then blocked with 4% BSA at 4 °C overnight. After washed three times with PBST, the membranes were incubated with AP-conjugated goat-anti-mouse Ig at 37 °C for 1 h, and placed into the substrate solution containing nitro blue tetrazolium and 5-bromo-4-chloro-3-indolyl phosphate substrates (NBT/BCIP, Sigma, St. Louis, MO, USA) for staining.

### 4.6. SDS-PAGE and Western Blotting

For western blotting assay, the purified rVAP and LCDV particles were mixed with an equal volume of protein loading buffer separately and denatured for 5 min in boiling water, then subjected to SDS-PAGE and transferred to PVDF membranes separately using transfer buffer (25 mM Tris, 192 mM Gly, 3.5 mM SDS). After the membranes were blocked with 4% BSA at 4 °C overnight and washed three times with PBST, the hybridoma culture supernatants were added as the primary antibody, and incubated at 37 °C for 1.5 h. Following a further wash with PBST, AP-conjugated goat-anti-mouse Ig was added as the secondary antibody, incubated and washed as above, and the membranes were then placed into substrate solution (100 mM NaCl, 100 mM Tris and 5 mM MgCl_2_, pH 9.5) containing NBT/BCIP for staining. Culture supernatants of MAbs against WSSV, instead of hybridoma culture supernatants, served as negative control.

### 4.7. Indirect Immunofluorescence Assay

The FG cells were seeded on the cover slips according to the method described by Wu et al. [45]. Briefly, aseptic circular cover slips (Solarbio, Beijing, China) were put in 24-well plates, and about 10^4^ cells were seeded into each well and grown into a monolayer. Thereafter, the cells were infected with 4 TCID_50_/mL LCDV at 22 °C for 2 h. After removing unbound virus particles and washing three times with PBS, the cells were cultured for 24 h in a maintenance medium containing 2% FBS. After washed again, the cells were fixed with 4% paraformaldehyde (Sangon Biotech, Shanghai, China) for 15 min at room temperature, followed by sequential incubation with hybridoma culture supernatants as a primary antibody and fluorescein isothiocyanate (FITC)-conjugated goat-anti-mouse Ig (Sigma, St. Louis, MO, USA) diluted 1:256 in PBS as a secondary antibody at 37 °C for 1 h in the dark. The cell nuclei were stained in blue with DAPI (Roche, Basel, Switzerland). Cells without LCDV infection served as negative controls. Finally, the cells were observed under a fluorescence microscope.

### 4.8. Preparation of Mice Ascites

After screened by ELISA, western blotting and IFA, the five positive hybridomas were cultured with GIT medium in 24-well plates at 37 °C supplied with 5% CO_2_ separately. Then, 10^6^ hybridomas were collected into a 1.5 mL centrifuge tube and washed with a 1 mL preheated 1640 medium at 37 °C, followed by centrifuging at 100× *g* for 5 min and resuspending with 500 μL 1640 medium. The hybridomas were intraperitoneally injected to the nine-week-old BALB/c mice, which had been given an injection of 500 μL liquid paraffin one week ago. About 10 days after injection, the mice were anesthetized with the ether, and the ascites with anti-32 kDa VAP MAbs was collected with a 1 mL syringe, placed at 4 °C overnight and centrifuged at 760× *g* for 10 min. After removing the lipids in the upper layer, the ascites was centrifuged at 12,000× *g* for 30 min again. Finally, the supernatants were collected and further purified by the caprylic acid-ammonium sulfate precipitation method [46], and then completely dialyzed in PBS at 4 °C and stored at −80 °C until use.

### 4.9. Neutralization Assay

To analyze the neutralizing ability of the five MAbs to LCDV infection, the purified ascites of the five MAbs was adjusted to a concentration of 0.16 µg/mL and 1.6 µg/mL and filtered through a 0.22 µm acrodisc syringe filter separately, and then incubated with LCDV at 4 TCID_50_/mL at 20 °C for 2 h. FG cells cultured in 25 cm^2^ culture flasks or 96-well plates were inoculated with 500 μL or 50 μL mixtures at 20 °C for 2 h, washed three times with MEM without FBS after the mixtures were removed, and cultured in MEM supplied with 2% FBS for another 48 h. Subsequently, the LCDV copy numbers of FG cells in 25 cm^2^ culture flasks were detected with real-time quantitative PCR as described previously [15], and the cytopathic CPE was monitored using an inverted microscope, while the cells cultured in 96-well plates were subjected to MTT assay. In another group, the five anti-32 kDa VAP MAbs at a concentration of 1.6 µg/mL were incubated with LCDV and inoculated FG cells; then, FG cells grown in 25 cm^2^ culture flasks were collected at 24 and 72 hpi, and the LCDV copy numbers and CPE were detected. The MAbs against WSSV with the same concentration, instead of the five MAbs, were used to incubate LCDV as positive controls. FG cells without treatment served as negative controls. MTT cell viability assay was conducted as following: FG cells in 96-well plates were incubated with 50 μL MTT (5 mg/mL, Sigma, St. Louis, MO, USA) per well at 22 °C for 4 h. Then, the supernatant was removed and 150 μL DMSO was added and incubated for 10 min. The absorbencies were measured with an automatic ELISA reader at 490 nm.

### 4.10. Statistics

All data were expressed as mean ± standard deviation. The statistical analysis was performed using GraphPad Prism 7.0 Software and one-way ANOVO. Differences were considered statistically significant for *p* < 0.05.
